# Mass Effect Score Associations in Comatose Trauma Patients With Intracranial Hemorrhage Requiring Mechanical Ventilation: A Retrospective Study

**DOI:** 10.7759/cureus.116058

**Published:** 2026-09-10

**Authors:** C. Michael Dunham, Gregory S Huang

**Affiliations:** 1 Trauma, Critical Care, and General Surgery Services, St. Elizabeth Youngstown Hospital, Youngstown, USA

**Keywords:** marshall ct score, mass effect score, mechanical ventilation, retrospective study, rotterdam ct score, trauma centers, traumatic intracranial hemorrhage

## Abstract

Objective

There is a paucity of studies evaluating computed tomography (CT) scores in patients with Glasgow Coma Scale (GCS) scores of 3-8. This study assessed the associations between risk factors (including admission GCS score, head Abbreviated Injury Scale (AIS) score, brain mass effect score (MES), and age) and unfavorable outcomes (including in-hospital mortality, adverse outcomes, failure to follow commands at hospital discharge, and failure to follow commands at three months) in mechanically ventilated patients with intracranial hemorrhage (ICH) and GCS scores of 3-8. In relation to these outcomes, we also compared the MES with the Marshall (MAR) classification and Rotterdam (ROT) score.

Methods

The adult blunt trauma cohort had ICH, GCS 3-8, need for mechanical ventilation, and level I trauma center admission (2018-2024). The GCS, age, AIS, ventilator status, intensive care unit (ICU) stay, hospital stay, and hospital mortality were obtained from the trauma registry. Adverse outcomes included hospital death, ICU stay ≥18 days, or hospital stay >20 days. MES indicated the presence (1) or absence (0) for each of the following: basal cistern compression, midline shift, and lateral ventricle compression (0-3). Brain CT images were reviewed to compute MES, MAR, and ROT. Electronic medical records (EMRs) were reviewed to determine following command status at hospital discharge and at three months after injury.

Results

Among the 148 blunt trauma patients, univariate associations with mortality were MES (p < 0.01), GCS (p = 0.46), age ≥70 (p < 0.01), and AIS (p < 0.01); multivariate associations were MES (p < 0.01), age ≥70 (p < 0.01), and AIS (p > 0.10). For adverse outcomes, univariate associations were MES (p < 0.01), GCS (p = 0.46), age ≥70 (p = 0.04), and AIS (p < 0.01); multivariate associations were MES (p < 0.01), age ≥70 (p > 0.05), and AIS (p > 0.05). For failure to follow commands at discharge, univariate associations were MES (p < 0.01), GCS (p = 0.75), age (p = 0.01), and AIS (p < 0.01); multivariate associations were MES (p < 0.01), age (p = 0.01), and AIS (p > 0.10). For failure to follow commands at three months, univariate associations were MES (p < 0.01), GCS (p = 0.30), age (p < 0.01), and AIS (p < 0.01); multivariate associations were MES (p < 0.01), age (p < 0.01), and AIS (p > 0.10). MES correlated strongly with MAR (p < 0.01; r = 0.83) and ROT (p < 0.01; r = 0.82). In multivariate stepwise continuous score regression (MCR), mortality and adverse outcome were associated with MES (both p < 0.01), but not MAR or ROT (both p > 0.05). For failure to follow commands at discharge and three months, MCR showed an association with ROT (both p < 0.01), but not MES or MAR (both p > 0.05). In multivariate stepwise dichotomous score regression (MDR), mortality and adverse outcomes were associated with MES ≥2 (both p < 0.01), but not MAR ≥4 or ROT ≥4 (both p > 0.05). Failure to follow commands at discharge was associated with ROT ≥4 (p < 0.01), but not MES ≥2 or MAR ≥4 (p > 0.05). Failure to follow commands at three months was associated with MES ≥2 (p < 0.01), but not MAR ≥4 or ROT ≥4 (p > 0.05).

Conclusions

MES was a better predictor of adverse outcomes than AIS and GCS in comatose patients with ICH. MES was competitive with other established brain CT scores for predicting adverse outcomes. Further studies are needed to confirm these findings.

## Introduction

A dozen studies have demonstrated that brain computed tomography (CT) scores can prognosticate hospital and post-discharge outcomes in patients with traumatic brain injury [1-12]. The typical brain CT scoring methods used in these studies have been the Marshall (MAR) classification system [1-3,7-11] and the Rotterdam (ROT) scoring system [1-7,9,10,12]. We have developed an intracranial hemorrhage (ICH) mass effect score (MES) that considers the presence of a midline shift [2,10,12], basal cistern compression [2,10,12], and lateral ventricular compression [13-15]. Additionally, we have shown that this ICH MES is associated with the need for surgical decompression of ICH [16,17] and with unfavorable hospital and post-discharge outcomes [18,19]. While most studies investigating the MAR and ROT scoring systems have included patients with Glasgow Coma Scale (GCS) scores of 3-12 or 3-15, only three studies have assessed patients with traumatic brain injury and GCS scores of 3-5 [2] or 3-8 [10,12].

Abbreviated Injury Scale (AIS) scores are recognized as a mechanism for stratifying injuries in multiple body regions according to severity [20]. Multiple investigators have reported that head AIS scores are associated with hospital mortality in trauma patients [21-23]. Researchers have also demonstrated that head AIS scores are higher in trauma patients with prolonged intensive care unit (ICU) stays [22] and in those with unfavorable 12-month GCS scores [24]. The magnitude of the ICH is a principal element in traumatic brain injury when assigning the head AIS [20,25-27]. However, these same authors stipulate that the presence of fractures and/or altered levels of consciousness may also be used when assigning the head AIS. From a contextual standpoint, each type of ICH (e.g., subdural hematoma and cerebral contusion) has a family of relevant AIS codes. From that group of codes, a specific AIS code and its corresponding AIS score are selected by the trauma registry coder based on the magnitude of the ICH described in the CT report (tiny, small, moderate, large, or massive). Thus, the head AIS may be based on a categorical description of the size of the ICH as portrayed in the CT report. We previously performed a trauma registry audit to assess each AIS code used to determine the head AIS score for 173 patients with ICH who underwent magnetic resonance imaging [19]. The auditors found that more than 90% of the highest head AIS codes were based on CT characteristics of the intracranial macrohemorrhage. Based on this audit, the highest head AIS is referred to as the ICH AIS in this manuscript. We have previously demonstrated that the ICH AIS in blunt trauma patients with ICH is associated with unfavorable hospital and post-injury outcomes [18,19,28].

Given the relative paucity of CT scoring studies in patients with GCS scores of 3-8, we investigated the effectiveness of our ICH MES in prognosticating unfavorable hospital and post-discharge outcomes in adult blunt trauma patients with coma (GCS 3-8), ICH, and a need for mechanical ventilation. The four unfavorable outcomes were hospital death; adverse outcomes (hospital death, ICU stay ≥18 days, or hospital stay >20 days); failure to follow commands at hospital discharge; and failure to follow commands at three months. The first aim was to assess the associations of risk conditions (trauma mechanism, extracranial (EC) injury severity, craniotomy, GCS, age, type of ICH, ICH AIS, and ICH MES) with each unfavorable outcome. The second objective was to determine whether the ICH MES would outperform or be comparable to ROT scores and MAR classification levels in their relationships with each of the four unfavorable outcomes.

## Materials and methods

Ethics statement

This study was reviewed by the local institutional review board (IRB) and was determined to qualify for exemption under Exempt Category 4 (Bon Secours Mercy Health, IRB number 2026-Trauma-Dunham5). The requirement for informed consent was waived because of the minimal risk and retrospective nature of the investigation. All medical records were de-identified.

Study design and population

In this retrospective study, the parent group consisted of adult blunt trauma patients with ICH who were admitted to a level I trauma center in Northeast Ohio. The study periods were January 21 to July 21 in 2018, 2019, and 2020, and January 1 to December 31 in 2021, 2022, 2023, and 2024. The partial-year data for 2018-2020 were obtained from a database used for a previous publication that examined the Ohio stay-at-home order for coronavirus disease 2019 (COVID-19) [29]. The current study represents a subset of the cohort described in a previous publication that included all patients with blunt trauma, ICH, and GCS scores of 3-15 who were admitted to the trauma center during these seven years [28].

Inclusion and exclusion criteria

Data obtained from the trauma registry were used to identify the current study cohort, consisting of trauma center admissions meeting the following criteria: age ≥18 years, blunt trauma, ICH, admission GCS score of 3-8, and need for mechanical ventilation. Exclusion criteria were age <18 years, a non-blunt mechanism of injury, absence of ICH, GCS score >8, no requirement for mechanical ventilation, or no admission to the trauma center.

Data collection

Trauma Registry Data Source

Data from the trauma registry included age, GCS [17-19,28,30-32], Injury Severity Score (ISS) [18,19,28,31-33], highest head AIS [18-20,28], hospital mortality, ICU stay (days), hospital stay (days), and need for mechanical ventilation. Because a trauma registry audit demonstrated that the highest head AIS was directly related to the magnitude of the ICH in >90% of patients, the highest head AIS is subsequently referred to as the ICH AIS [18,19,28]. The EC-ISS was calculated as follows: (total ISS) - (ICH AIS)². This calculation has been shown to have univariate and multivariate associations with unfavorable outcomes [18,19,28].

Risk conditions

Risk conditions considered were trauma mechanism (ground-level fall or other blunt trauma mechanism), decreased GCS, increased ICH AIS, increased EC-ISS, increased age, need for craniotomy, subarachnoid hemorrhage, intracerebral hemorrhage (cerebral contusion or hematoma), subdural hematoma, epidural hematoma, and increased brain CT scores (MAR classification levels, ROT scores, and the study ICH MES).

The MAR classification levels are mutually exclusive and progress as follows: no visible pathology, level 1; ICH ≤25 cm³ (≤25 mL), level 2; basal cistern compression, level 3; midline shift >5 mm, level 4; ICH with surgical evacuation, level 5; and high- or mixed-density lesions >25 cm³ (>25 mL) (intra- or extra-axial) without evacuation, level 6. The range is 1-6, with a worrisome score defined as ≥4 [1-3,7-11]. The ROT scores are assigned as follows: basal cistern compression, 1 point; absent basal cisterns, 2 points; midline shift >5 mm, 1 point; intraventricular or subarachnoid hemorrhage, 1 point; epidural hematoma absent, 1 point; and 1 point routinely added to the aforementioned points. The range is 1-6, with a worrisome score defined as ≥4 [1-7,9,10,12]. The ICH MES is calculated as follows: bilateral or unilateral lateral ventricular compression (1 = yes) + midline shift (1 = yes) + basal cistern compression (1 = yes). The score ranges from 0 to 3, with a worrisome score defined as ≥2 [16-19].

Using the EMR, the first author reviewed the CT images obtained during the first 18 hours following brain trauma and identified the most worrisome brain CT for each patient. Each selected CT was scored according to the MAR, ROT, and ICH MES methodologies without consideration of other risk conditions or undesirable outcomes. The first author also recorded whether the CT demonstrated an intracerebral hemorrhage, subarachnoid hemorrhage, subdural hematoma, or epidural hematoma. Because the imaging components of the brain CT scoring systems are commonly known, representative images are not included in the manuscript.

All brain CT images of study patients were generated using a General Electric Medical Systems or Siemens SOMATOM scanner (Siemens Healthineers AG, Erlangen, Germany) with 128 detector rows, a helical imaging technique, and a rotation time of 0.23-0.35 seconds. The software used to assess the images was The Change Healthcare Radiology Solutions Radiology Station Lite (version 2.0, Change Healthcare Canada Company, Nashville, TN, USA).

The trauma mechanism had previously been delineated in the parent group of patients with blunt trauma, ICH, and GCS scores of 3-15 as either a ground-level fall or another blunt trauma mechanism based on an EMR review [28].

Undesirable or poor outcomes

The trauma registry supplied information regarding hospital death, ICU stay (days), and hospital stay (days). Previously, we defined adverse outcomes as hospital death, ICU stay ≥2 days, or hospital stay >5 days [18,19]. However, those investigations included blunt trauma patients with ICH and GCS scores of 3-15. Because the current cohort of patients with ICH had GCS scores of 3-8, and the upper 75th percentile cutoff points for ICU stay and hospital stay were 15 and 19 days, respectively, we modified the criteria for adverse outcomes. In the current investigation, the adverse outcome was defined as hospital death, ICU stay ≥18 days, or hospital stay >20 days.

Patients were classified as not following commands at hospital discharge if (1) they died during the trauma center stay, as documented in the trauma registry, or (2) the EMR of hospital survivors demonstrated that they were not following commands at hospital discharge [16,17].

Patients were categorized as not following commands at three months if (1) they died during the trauma center stay, as documented in the trauma registry; (2) a hospital survivor died within three months following hospital discharge, as documented in the EMR; or (3) they were alive at three months but were not following commands, as documented in the EMR [16,17].

Thus, the four undesirable outcomes were hospital death, adverse outcomes, not following commands at hospital discharge, and not following commands at three months.

Statistical analysis

Continuous data without a normal distribution (age) and ordinal data (e.g., GCS, ICH AIS, MAR levels, ROT scores, and ICH MES) are presented as the median and interquartile range. Dichotomous proportional data (e.g., hospital death, adverse outcome, not following commands at hospital discharge, not following commands at three months, ground-level fall mechanism, EC-ISS ≥6, age ≥70 years, craniotomy, intracerebral hemorrhage, subarachnoid hemorrhage, subdural hematoma, epidural hematoma, MAR level ≥4, ROT score ≥4, and ICH MES ≥2) are presented as counts (N) and percentages.

For dichotomous proportional data conforming to a 2 × 2 contingency table, the chi-square test was used for univariate analysis to calculate p-values and chi-square values. Odds ratios and risk ratios were calculated to quantify proportional differences, when appropriate. When comparing median values between two independent groups, the Wilcoxon rank-sum test was used to assess univariate associations. A Spearman correlation coefficient was calculated to assess the relationships between ICH MES and ICH AIS, MAR classification levels, and ROT scores.

Because the four undesirable outcomes were dichotomous, stepwise multivariate analyses were performed using logistic regression to assess independent associations. Risk conditions (ground-level fall mechanism, EC-ISS ≥6, age ≥70 years, craniotomy, intracerebral hemorrhage, subarachnoid hemorrhage, subdural hematoma, epidural hematoma, MAR level ≥4, ROT score ≥4, ICH MES ≥2, GCS, ICH AIS, MAR levels, ROT scores, and ICH MES) with a univariate association (p ≤ 0.1000) for a given undesirable outcome were included in the stepwise regression analysis for that particular outcome.

Data were entered into Excel 2010 (Microsoft Corporation, Redmond, WA, USA) and imported into SAS System for Windows (release 9.2; SAS Institute Inc., Cary, NC, USA). The significance level was set at p < 0.05.

## Results

The parent group included 1,545 blunt trauma patients with ICH and GCS scores of 3-15. The current study cohort consisted of 148 blunt trauma patients who required ICU admission and mechanical ventilation and had GCS scores of 3-8. Among these 148 patients, 19 (12.8%) were treated during the Ohio stay-at-home order for COVID-19. Risk conditions and outcome results are presented in Table 1. Most patients had an admission GCS score of 3-5. The proportions of patients with worrisome ICH MES, MAR classification levels, and ROT scores were virtually identical. Hospital death occurred in nearly one-third of the patients. Among patients admitted during the Ohio stay-at-home order for COVID-19, the proportion with an ICH MES ≥2 was 8/19 (42.1%).

**Table 1:** Risk condition and outcome results for all patients ICH: intracranial hemorrhage; GCS: Glasgow Coma Scale; AIS: Abbreviated Injury Scale; EC-ISS: extracranial Injury Severity Score; IQR: interquartile range.

Variable	Result
Number	148 (100%)
Ground-level fall mechanism (no.; percent)	37 (25.0%)
Subarachnoid hemorrhage (no.; percent)	122 (82.4%)
Cerebral contusion or hematoma (no.; percent)	71 (48.0%)
Subdural hematoma (no.; percent)	97 (65.5%)
Epidural hematoma (no.; percent)	13 (8.8%)
Craniotomy (no.; percent)	34 (23.0%)
Study ICH mass effect score ≥2 (no.; percent)	63 (43.9%)
Marshall level ≥4 (no.; percent)	64 (43.2%)
Rotterdam score ≥4 (no.; percent)	70 (47.3%)
GCS 3-5 (no.; percent)	109 (73.7%)
Age (median; IQR)	54.5 (33-69)
ICH AIS ≥4 (no.; percent)	122 (82.4%)
EC–ISS ≥6 (no.; percent)	74 (50.0%)
Hospital death (no.; percent)	47 (31.8%)
Intensive care unit days (median; IQR)	11 (6-16)
Hospital days (median; IQR)	13 (8-19)
Adverse outcome (no.; percent)	79 (53.4%)
Not following commands at discharge (no.; percent)	82 (55.4%)
Not following commands at three months (no.; percent)	61 (41.2%)
Required mechanical ventilation (no.; percent)	148 (100%)

Univariate associations between risk conditions and death are presented in Table 2. ICH MES, age ≥70 years, and ICH AIS demonstrated significant univariate associations with death. ICH AIS had a moderate correlation with ICH MES (p < 0.0001; r = 0.49). Multivariate logistic regression analysis demonstrated that death was independently associated with increased ICH MES (p < 0.0001) and age ≥70 years (p = 0.0001), but not with increased ICH AIS (p > 0.1000).

**Table 2:** Risk conditions for patients with hospital death ICH: intracranial hemorrhage, IQR: interquartile range, GCS: Glasgow Coma Scale, AIS: Abbreviated Injury Scale, EC-ISS, extracranial Injury Severity Score.

Variable	Lived	Died	Wilcoxon p-value	χ^2^ p-value	χ^2^ value
Number	101 (68.2%)	47 (31.8%)	–	–	–
Ground-level fall mechanism	22 (21.8%)	15 (31.9%)	–	0.1851	1.8
ICH mass effect score (median; IQR)	1 (0-2)	3 (2-3)	<0.0001	–	–
ICH mass effect score ≥2	29 (28.7%)	36 (76.6%)	–	<0.0001	29.9
GCS (median; IQR)	3 (3-6)	3 (3-5)	0.4612	–	–
Age (median; IQR)	50 (31-61)	67 (49-78)	0.0003	–	–
Age ≥70 years	14 (13.9%)	21 (44.7%)	–	<0.0001	16.9
ICH AIS (median; IQR)	4 (4-5)	5 (4-5)	0.0004	–	–
EC-ISS (median; IQR)	8 (1-13)	4 (0-13)	0.3706	–	–
Craniotomy	21 (20.8%)	13 (27.7%)	–	0.3552	0.9

Univariate associations between risk conditions and adverse outcome are presented in Table 3. ICH MES, age ≥70 years, and ICH AIS demonstrated significant univariate associations with adverse outcomes. Multivariate logistic regression analysis demonstrated that the adverse outcome was independently associated with increased ICH MES (p < 0.0001), but not with increased ICH AIS (p > 0.0500) or age ≥70 years (p > 0.0500).

**Table 3:** Risk conditions for patients with adverse outcomes ICH: intracranial hemorrhage, IQR: interquartile range, GCS: Glasgow Coma Scale, AIS: Abbreviated Injury Scale, EC-ISS: extracranial Injury Severity Score.

Variable	No adverse outcomes	With adverse outcomes	Wilcoxon p-value	χ^2^ p-value	χ^2^ value
Number	69 (46.6%)	79 (53.4%)	–	–	–
Ground-level fall mechanism	17 (24.6%)	20 (25.3%)	–	0.9242	0.1
ICH mass effect score (median; IQR)	0 (0-1)	2 (1-3)	<0.0001	–	–
ICH mass effect score ≥2	16 (23.2%)	49 (62.0%)	–	<0.0001	22.6
GCS (median; IQR)	3 (3-7)	3 (3-5)	0.4580	–	–
Age (median; IQR)	52 (30-65)	57 (35-74)	0.1318	–	–
Age ≥70 years	11 (15.9%)	24 (30.4%)	–	0.0392	4.3
ICH AIS (median; IQR)	4 (4-4)	5 (4-5)	<0.0001	–	–
EC-ISS (median; IQR)	5 (1-13)	8 (1-18)	0.5661	–	–
Craniotomy	12 (17.4%)	22 (27.9%)	–	0.1314	2.3

Univariate associations between risk conditions and not following commands at hospital discharge are presented in Table 4. ICH MES, increased age, and ICH AIS demonstrated significant univariate associations with not following commands at hospital discharge. Multivariate logistic regression analysis demonstrated that not following commands at hospital discharge was independently associated with increased ICH MES (p < 0.0001) and increased age (p = 0.0140), but not with increased ICH AIS (p > 0.1000).

**Table 4:** Risk conditions for patients not following commands at hospital discharge ICH: intracranial hemorrhage, IQR: interquartile range, GCS: Glasgow Coma Scale, AIS: Abbreviated Injury Scale, EC-ISS: extracranial Injury Severity Score.

Variable	Following discharge commands	Not following discharge commands	Wilcoxon p-value	χ^2^ p-value	χ^2^ value
Number	66 (44.6%)	82 (55.4%)	–	–	–
Ground-level fall mechanism	17 (25.8%)	20 (24.4%)	–	0.8486	0.1
ICH mass effect score (median; IQR)	0 (0-1)	2 (0-3)	<0.0001	–	–
ICH mass effect score ≥2	15 (22.7%)	50 (61.0%)	–	<0.0001	21.7
GCS (median; IQR)	3 (3-5)	3 (3-6)	0.7539	–	–
Age (median; IQR)	48 (29-61)	59 (37-73)	0.0077	–	–
Age ≥70 years	10 (15.2%)	25 (30.5%)	–	0.0291	4.8
ICH AIS (median; IQR)	4 (4-5)	5 (4-5)	0.0003	–	–
EC-ISS (median; IQR)	8 (1-15)	5 (1-13)	0.8109	–	–
Craniotomy	13 (19.7%)	21 (25.6%)	–	0.3953	0.7

Univariate associations between risk conditions and not following commands at three months are presented in Table 5. ICH MES, increased age, and ICH AIS demonstrated significant univariate associations with not following commands at three months. Multivariate logistic regression analysis demonstrated that not following commands at three months was independently associated with increased ICH MES (p < 0.0001) and increased age (p = 0.0001), but not with increased ICH AIS (p > 0.1000).

**Table 5:** Risk conditions for patients not following commands at three months ICH: intracranial hemorrhage, IQR: interquartile range, GCS: Glasgow Coma Scale, AIS: Abbreviated Injury Scale, EC-ISS: extracranial Injury Severity Score.

Variable	Following commands at three months	Not following commands at three months	Wilcoxon p-value	χ^2^ p-value	χ^2^ value
Number	87 (58.8%)	61 (41.2%)	–	–	–
Ground-level fall mechanism	19 (21.8%)	18 (29.5%)	–	0.2889	1.1
ICH mass effect score (median; IQR)	1 (0-2)	3 (1-3)	<0.0001	–	–
ICH mass effect score ≥2	24 (27.6%)	41 (67.2%)	–	<0.0001	22.9
GCS (median; IQR)	3 (3-7)	3 (3-5)	0.2998	–	–
Age (median; IQR)	47 (29-61)	62 (51-75)	<0.0001	–	–
Age ≥70 years	12 (13.8%)	23 (37.7%)	–	0.0008	11.4
ICH AIS (median; IQR)	4 (4-5)	5 (4-5)	0.0010	–	–
EC-ISS (median; IQR)	8 (1-15)	5 (1-13)	0.7667	–	–
Craniotomy	18 (20.7%)	16 (26.2%)	–	0.4303	0.6

The proportion of patients undergoing craniotomy was greater among those with an epidural hematoma (61.5%) than among those without an epidural hematoma (19.3%; p = 0.0005; odds ratio = 6.7). Median GCS scores were the same among those with an epidural hematoma (3.0) and those with other types of ICH (3.0; Wilcoxon p = 0.1884). Univariate associations between ICH categories and hospital death are presented in Table 6. Subdural hematoma had a nearly significant univariate association with hospital death. Multivariate logistic regression analysis demonstrated that hospital death was independently associated with increased ICH MES (p < 0.0001) and absence of an epidural hematoma (p = 0.0184). Other ICH categories had no independent associations with hospital death (p > 0.1000).

**Table 6:** Associations of intracranial hemorrhage categorizations with hospital death

Variable	Lived	Died	χ^2^ p-value	χ^2^ value
Number	101 (68.2%)	47 (31.9%)	–	–
Subarachnoid hemorrhage	82 (81.2%)	40 (85.1%)	0.5598	0.3
Cerebral hematoma or contusion	44 (43.6%)	27 (57.5%)	0.1155	2.5
Subdural hematoma	61 (60.4%)	36 (76.6%)	0.0535	3.7
Epidural hematoma	11 (10.9%)	2 (4.3%)	0.1843	1.8

Univariate associations between ICH categories and adverse outcomes are presented in Table 7. Subdural hematoma demonstrated a significant univariate association with adverse outcomes. Multivariate logistic regression analysis demonstrated that adverse outcomes were independently associated with increased ICH MES (p < 0.0001). No ICH category had an independent association with adverse outcomes (p > 0.0500).

**Table 7:** Associations of intracranial hemorrhage categorizations with adverse outcomes

Variable	No adverse outcomes	With adverse outcomes	χ^2^ p-value	χ^2^ value
Number	69 (46.6%)	79 (53.4%)	–	–
Subarachnoid hemorrhage	55 (79.7%)	67 (84.8%)	0.4160	0.7
Cerebral hematoma or contusion	30 (43.5%)	41 (51.9%)	0.3064	1.1
Subdural hematoma	39 (56.5%)	58 (73.4%)	0.0310	4.7
Epidural hematoma	7 (10.1%)	6 (7.6%)	0.5846	0.3

Univariate associations between ICH categories and not following commands at hospital discharge are presented in Table 8. Subdural hematoma and intracerebral hemorrhage demonstrated significant univariate associations with not following commands at hospital discharge. Multivariate logistic regression analysis demonstrated that not following commands at hospital discharge was independently associated with increased ICH MES (p < 0.0001) and absence of an epidural hematoma (p = 0.0192). Other ICH categories had no independent associations with not following commands at hospital discharge (p > 0.0500).

**Table 8:** Associations of intracranial hemorrhage categorizations with not following commands at hospital discharge

Variable	Following discharge commands	Not following discharge commands	χ^2^ p-value	χ^2^ value
Number	66 (44.6%)	82 (55.4%)	–	–
Subarachnoid hemorrhage	52 (78.8%)	70 (85.4%)	0.2959	1.1
Cerebral hematoma or contusion	24 (36.4%)	47 (57.3%)	0.0112	6.4
Subdural hematoma	36 (54.6%)	61 (74.4%)	0.0116	6.4
Epidural hematoma	8 (12.1%)	5 (6.1%)	0.1981	1.7

Univariate associations between ICH categories and not following commands at three months are presented in Table 9. Epidural and subdural hematoma categories demonstrated significant univariate associations with not following commands at three months. Multivariate logistic regression analysis demonstrated that not following commands at three months was independently associated with increased ICH MES (p < 0.0001) and absence of an epidural hematoma (p = 0.0016). Other ICH categories had no independent associations with not following commands at three months (p > 0.0500).

**Table 9:** Associations of intracranial hemorrhage categorizations with not following commands at three months

Variable	Following commands at three months	Not following commands at three months	χ^2^ p-value	χ^2^ value
Number	87 (58.8%)	61 (41.2%)	–	–
Subarachnoid hemorrhage	69 (79.3%)	53 (86.9%)	0.2333	1.4
Cerebral hematoma or contusion	36 (41.4%)	35 (57.4%)	0.0552	3.7
Subdural hematoma	51 (58.6%)	46 (75.4%)	0.0344	4.5
Epidural hematoma	11 (12.6%)	2 (3.3%)	0.0476	3.9

ICH MES demonstrated strong correlations with MAR classification levels (p < 0.0001; r = 0.83) and ROT scores (p < 0.0001; r = 0.82).

Comparisons of the three CT scores in relation to death are presented in Table 10. The chi-square, odds ratio, and risk ratio values were highest for the ICH MES.

**Table 10:** Computed tomography score comparisons for hospital death ICH: intracranial hemorrhage.

Variable	Lived	Died	Wilcoxon p-value	χ^2^ p-value	χ^2^ value	OR	RR
Number	101 (68.2%)	47 (31.9%)	–	–	–	–	–
ICH mass effect score (median; IQR)	1 (0-2)	3 (2-3)	<0.0001	–	–	–	–
Marshall level (median; IQR)	2 (2-4)	5 (3-6)	<0.0001	–	–	–	–
Rotterdam score (median; IQR)	3 (3-4)	4 (4-5)	<0.0001	–	–	–	–
ICH mass effect score ≥2	29 (28.7%)	36 (76.6%)	–	<0.0001	29.9	8.1	2.7
Marshall level ≥4	31 (30.7%)	33 (70.2%)	–	<0.0001	20.4	5.3	2.3
Rotterdam score ≥4	33 (32.7%)	37 (78.7%)	–	<0.0001	27.3	7.6	2.4

Comparisons of the three CT scores in relation to adverse outcomes are presented in Table 11. The chi-square, odds ratio, and risk ratio values were highest for the ICH MES.

**Table 11:** Computed tomography score comparisons for adverse outcomes ICH: intracranial hemorrhage.

Variable	No adverse outcomes	With adverse outcome	Wilcoxon p-value	χ^2^ p-value	χ^2^ value	OR	RR
Number	69 (46.6%)	79 (53.4%)	–	–	–	–	–
ICH mass effect score (median; IQR)	0 (0-1)	2 (1-3)	<0.0001	–	–	–	–
Marshall level (median; IQR)	2 (2-4)	4 (2-5)	<0.0001	–	–	–	–
Rotterdam score (median; IQR)	3 (3-4)	4 (3-5)	<0.0001	–	–	–	–
ICH mass effect score ≥2	16 (23.2%)	49 (62.0%)	–	<0.0001	22.6	5.4	2.7
Marshall level ≥4	20 (29.0%)	44 (55.7%)	–	0.0011	10.7	3.1	1.9
Rotterdam score ≥4	19 (27.5%)	51 (64.6%)	–	<0.0001	20.3	4.8	2.3

Comparisons of the three CT scores in relation to not following commands at hospital discharge are presented in Table 12. The chi-square, odds ratio, and risk ratio values for the ICH MES were comparable to those for the ROT score but higher than those for the MAR classification.

**Table 12:** Computed tomography score comparisons for not following commands at hospital discharge ICH: intracranial hemorrhage.

Variable	Following discharge commands	Not following discharge commands	Wilcoxon p-value	χ^2^ p-value	χ^2^ value	OR	RR
Number	66 (44.6%)	82 (55.4%)	–	–	–	–	–
ICH mass effect score (median; IQR)	0 (0-1)	2 (0-3)	<0.0001	–	–	–	–
Marshall level (median; IQR)	2 (2-4)	4 (2-5)	0.0001	–	–	–	–
Rotterdam score (median; IQR)	3 (3-4)	4 (3-5)	<0.0001	–	–	–	–
ICH mass effect score ≥2	15 (22.7%)	50 (61.0%)	–	<0.0001	21.7	5.3	2.7
Marshall level ≥4	19 (28.8%)	45 (54.9%)	–	0.0014	10.1	3.0	1.9
Rotterdam score ≥4	17 (25.8%)	53 (64.6%)	–	<0.0001	22.2	5.3	2.5

Comparisons of the three CT scores in relation to not following commands at three months are presented in Table 13. The chi-square, odds ratio, and risk ratio values for the ICH MES were comparable to those for the ROT score but higher than those for the MAR classification.

**Table 13:** Computed tomography score comparisons for not following commands at three months ICH: intracranial hemorrhage.

Variable	Following commands at three months	Not following commands at three months	Wilcoxon p-value	χ^2^ p-value	χ^2^ value	OR	RR
Number	87 (58.8%)	61 (41.2%)	–	–	–	–	–
ICH mass effect score (median; IQR)	1 (0-2)	3 (1-3)	<0.0001	–	–	–	–
Marshall level (median; IQR)	2 (2-4)	4 (2-5)	<0.0001	–	–	–	–
Rotterdam score (median; IQR)	3 (3-4)	4 (3-5)	<0.0001	–	–	–	–
ICH mass effect score ≥2	24 (27.6%)	41 (67.2%)	–	<0.0001	22.9	5.4	2.4
Marshall level ≥4	27 (31.0%)	37 (60.7%)	–	0.0003	12.8	3.4	2.0
Rotterdam score ≥4	27 (31.0%)	43 (70.5%)	–	<0.0001	22.4	5.3	2.3

Table 14 displays the best continuous CT score predictor for the undesirable outcomes, as determined by multivariate stepwise regression analysis. The ICH MES was selected for two outcomes (death and adverse outcomes), whereas the ROT score was selected for the other two outcomes (not following commands at hospital discharge and at three months).

**Table 14:** Best continuous computed tomography score associations using the multivariate stepwise regression analysis for undesirable outcomes ICH: intracranial hemorrhage.

CT variable	Death	Adverse outcomes	Not following discharge commands	Not following commands at three months
ICH mass effect score	p < 0.0001	p < 0.0001	p > 0.0500	p > 0.0500
Marshall level	p > 0.0500	p > 0.0500	p > 0.0500	p > 0.0500
Rotterdam score	p > 0.0500	p > 0.0500	p < 0.0001	p < 0.0001

Table 15 displays the best dichotomous CT score predictor for the undesirable outcomes, as determined by multivariate stepwise regression analysis. The ICH MES was selected for three outcomes (death, adverse outcome, and not following commands at three months), whereas the ROT score was selected for the other outcome (not following commands at hospital discharge).

**Table 15:** Best dichotomous computed tomography score associations using the multivariate stepwise regression analysis for undesirable outcomes ICH: intracranial hemorrhage.

CT variable	Death	Adverse outcome	Not following discharge commands	Not following commands at three months
ICH mass effect score ≥2	p < 0.0001	p < 0.0001	p > 0.0500	p < 0.0001
Marshall level ≥4	p > 0.0500	p > 0.0500	p > 0.0500	p > 0.0500
Rotterdam score ≥4	p > 0.0500	p > 0.0500	p < 0.0001	p > 0.0500

## Discussion

Undesirable outcome associations with ICH MES, ICH AIS, age, and GCS

The current study demonstrates that the ICH MES had univariate and multivariate associations with all four undesirable outcomes (hospital death, adverse outcomes, not following commands at hospital discharge, and not following commands at three months). That is, the MES had associations with the three hospital outcomes and the one post-discharge outcome.

GCS had no significant univariate association with any of the four undesirable outcomes. We have demonstrated in earlier publications that a decreased GCS had associations with undesirable outcomes in patients with ICH; however, GCS scores were 3-15 [17-19,28]. Among the three published brain CT prediction studies in patients with traumatic brain injury coma, GCS was not described as having an association with an undesirable outcome in two investigations [2,10], but one study showed that GCS was significantly decreased with post-discharge unfavorable outcomes [12]. It is likely that the two publications, which did not mention a GCS association, did not observe a significant finding [2,10]. We believe that the absence of a univariate GCS association with any of the four undesirable outcomes strengthens the validity of the ICH MES.

It is essential to note that ICH AIS had a univariate association with all four undesirable outcomes. Because all four of the undesirable outcome multivariate analyses showed that the ICH AIS were insignificant, when considering the ICH MES, this fortifies the believability of the ICH MES. The modest correlation between ICH MES and ICH AIS supports the notion of our previous audit that head AIS in patients with ICH reflects, to some degree, the relative size of the ICH [19].

In the current study, increased age had a univariate association with all four undesirable outcomes and a multivariate association with three of the four outcomes. In earlier trauma publications, we have shown that increased age was associated with undesirable outcomes in cohorts where the ICH proportions were 100% [18] and 33% [31], respectively. Among the three published brain CT prediction studies in patients with traumatic brain injury coma, increased age was not described as having an association with an undesirable outcome in two investigations [2,10], but one study showed that age was significantly increased with post-discharge unfavorable outcomes [12]. Because the ICH MES had significant multivariate associations with all four undesirable outcomes, while simultaneously assessing increased age, this enhances the credibility of the ICH MES.

Undesirable outcome associations with ICH categories

The current study demonstrated that subarachnoid hemorrhage had no univariate association with any of the four undesirable outcomes. Relevant to these observations is that the subarachnoid hemorrhage proportion was >80% in the entire study cohort. Among the three published brain CT prediction studies in patients with traumatic brain injury coma, two reported the presence or absence of a subarachnoid hemorrhage association with their undesirable outcome [10,12], whereas the third did not [2]. Analogous to the current study, one of the coma investigations (GCS 3-8 and a subarachnoid hemorrhage proportion of 77%) also showed that there was no undesirable outcome association with subarachnoid hemorrhage in the total cohort [12]. However, a caveat was given that when a CT was performed ≤2 hours after injury, subarachnoid hemorrhage had an association with post-discharge unfavorable outcomes [12]. In the second coma study, Pargaonkar et al. provided evidence that subarachnoid hemorrhage occurred in 58% of patients and was associated with hospital mortality [10]. Among four other CT studies that included patients with GCS >8, three have shown that subarachnoid hemorrhage was associated with an undesirable outcome [1,8,11], whereas the other showed no difference [3]. Of the three studies showing a difference, the subarachnoid hemorrhage proportions ranged from 35.3% to 53.2%, thus creating a more favorable circumstance for detecting an association as compared with the 80% in the current study of patients with GCS 3-8. The relevance of subarachnoid hemorrhage to undesirable outcomes in comatose patients with brain injury seems to be unclear at present. The current study and that of Yu et al. [12], bearing their caveat, question its importance. It would likely have been insightful if Elkbuli et al. had reported on the proportion of subarachnoid hemorrhage and its association with undesirable outcomes in their study of patients with GCS 3-5 [2].

Subdural hematoma had a significant or nearly significant univariate association with all four undesirable outcomes. However, the chi-square values were low, likely because the proportion of subdural hematomas in the cohort was moderately high (nearly 70%). Among the three published brain CT prediction studies in patients with traumatic brain injury coma, two investigations did not report the subdural hematoma proportion or the association of subdural hematoma with their undesirable outcome [2,12]. The other study cited a subdural hematoma proportion of 54% and a hospital mortality that was lower than that for the total cohort [10]. Among three published brain CT prediction studies in patients with traumatic brain injury that included patients with GCS >8, undesirable outcomes were not associated with subdural hematoma in two investigations [3,8], whereas the other study reported a significant association with hospital mortality [1]. The current study and relevant literature indicate that the presence of a subdural hematoma, per se, has little relevance to subsequent undesirable outcomes, especially in patients with coma.

The current study demonstrated that epidural hematoma had no univariate association with the three undesirable hospital outcomes but showed an increased proportion in those following commands at three months. Among the three published brain CT prediction studies in patients with traumatic brain injury coma, two did not mention any association of epidural hematoma with undesirable outcomes [2,10]. The third investigation provided evidence that epidural hematoma had no association with post-discharge unfavorable outcomes when considering the entire cohort [12]. However, in a subset of patients who had a brain CT >2 hours after injury, they did demonstrate that epidural hematoma was associated with post-discharge unfavorable outcomes [12]. This is a controversial finding considering that the Helsinki score deducts three points when an epidural hematoma is identified on the brain CT and the ROT score adds one point when an epidural hematoma is not seen [7,10]. Among five published brain CT prediction studies in patients with traumatic brain injury that included patients with GCS >8, four showed that patients with epidural hematoma had a decrease in undesirable outcomes [1,4,8,11], and one demonstrated no association of epidural hematoma with hospital survival [3]. In one of the aforementioned studies, which found that epidural hematoma had a decrease in unfavorable outcomes in the univariate analysis, they showed that epidural hematoma had an increase in unfavorable outcomes in the multivariate analysis [4]. This observation is perplexing. In summary, the univariate influence of epidural hematoma on undesirable traumatic brain injury outcomes is conflicting and without a definitive conclusion.

The current study showed that intracerebral hemorrhage (cerebral contusion and/or intracerebral hematoma) had a significant association with not following commands at hospital discharge, a nearly significant association with not following commands at three months, and no association with hospital death or adverse outcome. Among two published brain CT prediction studies in patients with traumatic brain injury that included patients with GCS >8, one study reported that cerebral contusions were associated with hospital survival [3], and the other study showed that intracerebral hematoma had no association with post-discharge unfavorable outcomes [8]. The current study and the reported literature regarding the association of intracerebral hemorrhage with undesirable outcomes after traumatic brain injury are uncertain.

When the four ICH categories and the ICH MES were entered into the stepwise logistic regression analysis, increased ICH MES had an independent association with all four undesirable outcomes. The absence of an epidural hematoma also had an independent association with hospital death, not following commands at hospital discharge, and not following commands at three months. Otherwise, the ICH categories had no independent associations. These observations are consistent with epidural hematoma point assignments used in the Helsinki (3 points deducted when epidural hematoma is present) and ROT(1 point added when epidural is absent) scoring systems [7,10].

Comparisons of the three CT intracranial rating systems in the current study patients

Each patient in the current study had their brain CT rated according to the ICH MES, MAR, and ROT methodologies. The ICH MES had strong correlations with MAR classification levels and ROT scores. Common to all three scores are elements of midline shift and basal cistern compression [7,10]. Other investigators have provided multivariate analysis evidence that undesirable outcomes of traumatic brain injury have independent associations with midline shift [8,10] and basal cistern compression [8,10,12].

Relative to hospital death, chi-square, odds ratio, and risk ratio values were highest for the ICH MES when compared with MAR classification levels and ROT scores. Regarding adverse outcomes, chi-square, odds ratio, and risk ratio values were highest for the ICH MES when compared with MAR classification levels and ROT scores. Relative to not following commands at hospital discharge, the chi-square, odds ratio, and risk ratio values of the ICH MES were comparable to those of the ROT scores but better than those of the MAR classification levels. Concerning not following commands at three months, the chi-square, odds ratio, and risk ratio values of the ICH MES were comparable to those of the ROT scores but better than those of the MAR classification levels. Relative to predicting undesirable outcomes, these univariate results indicate that the ICH MES are comparable to ROT scores and MAR classification levels.

When applying multivariate stepwise regression to assess the best statistical predictor among the three continuous CT scores, the ICH MES was selected for hospital death and adverse outcomes, and the ROT score was preferred for not following commands at hospital discharge and at three months. When applying the multivariate stepwise regression analysis to assess the best statistical predictor among the three dichotomous CT scores, an ICH MES ≥2 was selected for hospital death, adverse outcome, and not following commands at three months, and the ROT score ≥4 was preferred for not following commands at hospital discharge. The multivariate results indicate that the ICH MES were comparable to ROT scores and MAR classification levels. Other statistical comparisons of predictability have shown that ROT scores were better than MAR classification levels [2], were slightly better [1,7,10], or were similar [3,9]. Other investigators have also used a MAR level ≥4 [2] and ROT score ≥4 [2,12] as discriminant classification cutoff points for delineating associations with undesirable outcomes. However, others have used different cutoff points as determined by using the receiver operating characteristic curve analysis [1,3].

Comparisons of the current study with other CT intracranial scoring system investigations in comatose patients with brain injury

The current study demonstrated that the ICH MES had univariate and multivariate associations with all four undesirable outcomes (hospital death, adverse outcomes, not following commands at hospital discharge, and not following commands at three months). That is, the MES had associations with the three hospital outcomes and the one post-discharge outcome. Among the three published brain CT prediction studies in patients with traumatic brain injury coma, the outcome associations were either hospital mortality [2,10] or post-discharge unfavorable outcomes [12], but not both. The study by Pargaonkar et al. also provides a table depicting death proportions at three months according to the various CT score levels but does not organize the data into dichotomous groups where measures (e.g., odds ratio) could have been used to assess associations [10]. Further, two investigations provided only univariate associations [2,10], whereas only one study showed multivariate associations [12].

The current study assessed the univariate and multivariate associations of the four ICH types with each of the four undesirable outcomes. Among the three published brain CT prediction studies in patients with traumatic brain injury coma, none reported on cerebral contusion or intracerebral hematoma associations with undesirable outcomes [2,10,12]. All patients in the current study had CT-documented ICH; however, based on the number of patients described with a MAR level 1 categorization, two of the CT score coma studies had ICH proportions of 95.3% [2] and 86.6% [10], respectively. The third coma investigation provided no MAR classification level data or an ICH proportion but only indicated that all patients had GCS 3-8 [12].

The largest study of the three coma CT prediction investigations consisted of 455 patients [12]. It is concerning that these patients emanated from a pool of 1,198 patients where 62.0% were excluded due to missing data. Although the current study has some periods excluded from years 2018 to 2020, this was due to an intentional study design and not because data were missing. Another concern is that one of the three coma CT prediction investigations only used receiver operating characteristic curve analysis to assess MAR and ROT system discrimination [10]. The areas under the curve were modest for the MAR (0.74) and ROT (0.75) systems [10]. No measure of association (e.g., odds ratio) was performed to provide readers with another perspective regarding score performance. However, we want to applaud all three coma CT prediction investigation groups of authors for their substantial contribution to the literature.

Limitations of the study

The main limitation of the current study is its retrospective design, which precludes definitive conclusions regarding causality. Review of the brain CT images and scoring, according to the three comparative methods, by a neuroradiologist would have enhanced the validity of the conclusions. Review of the images by a neuroradiologist to confirm the presence or absence of each ICH category (subarachnoid hemorrhage, cerebral hematoma or contusion, subdural hematoma, epidural hematoma) would also have improved the strength of the study findings. The lack of multiple brain CT interpreters and interobserver and intraobserver reliability are problematic. The relatively small size of the current study and the exclusion of other trauma center involvement are also weaknesses. The study findings are limited only to other GCS 3-8 patients who require mechanical ventilation.

## Conclusions

The ICH MES is a univariate and multivariate predictor for all four of the undesirable outcomes in comatose patients with ICH. Although the ICH AIS was a univariate predictor, it had no independent association when the ICH MES was included. The GCS had no univariate association for any of the four undesirable outcomes. The ICH MES was competitive with other recognized brain CT scores for predicting undesirable outcomes. Other investigations are needed to confirm these observations. Such investigations should be adequately powered, prospective, and include multiple trauma centers.
